# Quantitative Immunohistochemical Landscapes and Chemoimmunotherapy Outcomes of ASCL1, NEUROD1, POU2F3, and YAP1 in Pure Small-Cell Lung Cancer: A Pilot Study

**DOI:** 10.3390/jcm15166415

**Published:** 2026-08-19

**Authors:** Yasemin Aydinalp Camadan, Arzu Demir Ispir, Emine Kilic Bagir, Burak Mete, Hatice Asoglu, Mehmet Turker, Sendag Yaslikaya, Mehmet Mutlu Kidi, Sedat Biter, Esra Asarkaya, Suheda Atas Ipek, Tolga Koseci, Ertugrul Bayram, Berksoy Sahin, Derya Gumurdulu, Ismail Oguz Kara

**Affiliations:** 1 Department of Medical Oncology, Faculty of Medicine, Cukurova University, Adana 01330, Turkey; 2 Department of Pathology, Faculty of Medicine, Cukurova University, Adana 01330, Turkey; 3 Department of Public Health, Faculty of Medicine, Cukurova University, Adana 01330, Turkey; 4 Department of Medical Oncology, Adana City Training and Research Hospital, Adana 01260, Turkey; 5 Department of Medical Oncology, Giresun Training and Research Hospital, Giresun 28100, Turkey

**Keywords:** small-cell lung cancer, ASCL1, NEUROD1, POU2F3, YAP1, immunohistochemistry, intratumoral heterogeneity, chemoimmunotherapy, atezolizumab, real-world data

## Abstract

**Background:** Recent transcriptomic studies classify small-cell lung cancer (SCLC) into molecular subtypes driven by ASCL1, NEUROD1, POU2F3, and YAP1. This study evaluated an immunohistochemistry (IHC)-based subtyping framework using real-world data and assessed its ability to predict chemoimmunotherapy outcomes. **Methods:** Proteomic expression profiles were quantified by IHC in 100 patients with SCLC. Clinicopathological trajectories and overall survival (OS) were analyzed using a parsimonious multivariate Cox model designed to mitigate overfitting. **Results:** Significant subclonal heterogeneity was captured by co-dominant hybrid phenotypes, including SCLC-AN (9%) and SCLC-AP (5%). In the treatment-adjusted, parsimonious multivariate analysis, individual continuous biomarker expressions showed an independent association with ASCL1 percentage (HR = 1.011, *p* = 0.017). Traditional extensive-stage disease (HR = 3.801, 95% CI: 1.669–8.657, *p* = 0.001) and synchronous bone metastases (HR = 1.968, 95% CI: 1.074–3.604, *p* = 0.028) remained the only robust clinical predictors of poor OS. When adjusted for therapeutic interventions, first-line chemoimmunotherapy showed a strong protective numerical trend, reducing mortality risk by 49% (HR = 0.512, *p* = 0.060). Within the immunotherapy subgroup (*n* = 19), the NE-low group had a 100% response rate and a numerically longer median overall survival than the NE-high group (49.4 vs. 21.4 months; log-rank *p* = 0.080). **Conclusions:** Our pilot evaluation provides a clinically feasible routine IHC framework for characterizing subclonal mosaicism in SCLC.

## 1. Introduction

Small-cell lung cancer (SCLC) accounts for approximately 13–15% of all lung malignancies [1]. Characterized by rapid proliferation and early metastatic dissemination, nearly 70% of SCLC patients present with advanced disease at initial diagnosis [2]. SCLC has historically been evaluated as a uniform disease entity due to shared histological features under light microscopy, alongside near-universal inactivation of the *TP53* and *RB1* tumor suppressor genes [3]. Consequently, therapeutic strategies depended primarily on standard protocols stratified solely by clinical stage. However, in limited-stage SCLC (LS-SCLC), consolidation therapy with durvalumab following concurrent chemoradiotherapy has recently emerged as a new standard of care, significantly improving overall survival [4]. For extensive-stage SCLC (ES-SCLC), first-line systemic intervention involves platinum–etoposide chemotherapy combined with programmed cell death ligand 1 (PD-L1) inhibitors, such as durvalumab or atezolizumab. Despite high initial objective response rates, therapeutic resistance typically develops within months, and median overall survival remains short at 12–13 months for patients with ES-SCLC [5,6]. Therefore, characterizing the underlying biological and molecular heterogeneity of SCLC is critical to identify predictive biomarkers of immunotherapy response and develop personalized clinical treatments [7].

Recent advances have significantly altered the biological understanding of SCLC. Preclinical evaluations using cell lines, genetically engineered mouse models, and patient-derived xenografts demonstrate that SCLC can be classified into molecular subtypes driven by specific lineage transcription factors: Achaete-scute homolog 1 (ASCL1), neuronal differentiation factor 1 (NEUROD1), POU class 2 homeobox 3 (POU2F3), and Yes-associated protein 1 (YAP1) [8]. SCLC-A, the most prevalent subtype, is marked by ASCL1 dominance, distinct neuroendocrine features, and classical morphology, displaying high expression of *DLL3*, *BCL2*, synaptophysin, chromogranin A, and TTF-1 [9]. The NEUROD1-dominant subtype (SCLC-N) also exhibits pronounced neuroendocrine features, often correlates with variant cell morphology, and frequently displays *MYC* amplification [10]. Conversely, the SCLC-P subtype is characterized by POU2F3 expression, lacks neuroendocrine differentiation, and is derived from rare chemosensory tuft cells of the respiratory tract [11]. While a YAP1-linked fourth subtype was initially suggested, subsequent investigations did not confirm YAP1 as an independent, consistent lineage predictor. Gay et al. proposed an alternative subtype designated as SCLC-inflamed (SCLC-I), characterized by low expression of ASCL1, NEUROD1, and POU2F3 (“triple-negative”) instead of a distinct YAP1 subtype. SCLC-I exhibits an inflammatory gene expression signature marked by increased expression of *CD8A* and *CD8B*, indicative of cytotoxic T-cell infiltration, alongside high expression of *CD274* and *PDCD1* [12]. Single-cell RNA sequencing analyses support this classification, demonstrating that ASCL1, NEUROD1, and POU2F3 expressions are specific to malignant epithelial cells, whereas YAP1 expression is primarily detected in non-malignant stromal and normal epithelial populations [13]. Furthermore, post hoc analyses of the IMpower133 and CASPIAN clinical trials suggest that the SCLC-I subtype may derive a survival benefit from the addition of immune checkpoint inhibitors to first-line chemotherapy [14].

While molecular subtyping via key transcription factors provides translational insights, classification has historically relied on RNA transcriptomic sequencing profiles. However, high-throughput gene expression profiling remains limited in routine clinical workflows due to financial constraints and tissue availability, making immunohistochemistry (IHC) a cost-effective and pragmatic alternative in pathology laboratories [15]. Nonetheless, discrepancies between transcriptomic findings and proteomic levels, the lack of standardized cut-off thresholds for defining IHC positivity, and the clinical relevance of multi-marker co-expression profiles influenced by intratumoral heterogeneity remain to be fully characterized [1,16,17,18]. Additionally, the non-validation of YAP1 as an independent lineage predictor and its potential role as a surrogate marker of the microenvironment present operational challenges in identifying specific subclonal subsets [7].

In this pilot study, the proteomic expression patterns of ASCL1, NEUROD1, POU2F3, and YAP1 were evaluated via quantitative IHC in a cohort of 100 patients with histologically confirmed pure SCLC. The primary objective of this research was to investigate the baseline association of these quantitative biomarker profiles with clinicopathological characteristics, long-term survival trajectories, and objective clinical response outcomes following first-line systemic chemoimmunotherapy regimens within a real-world setting.

## 2. Materials and Methods

### 2.1. Sample Selection and Study Design

**Figure 1:**
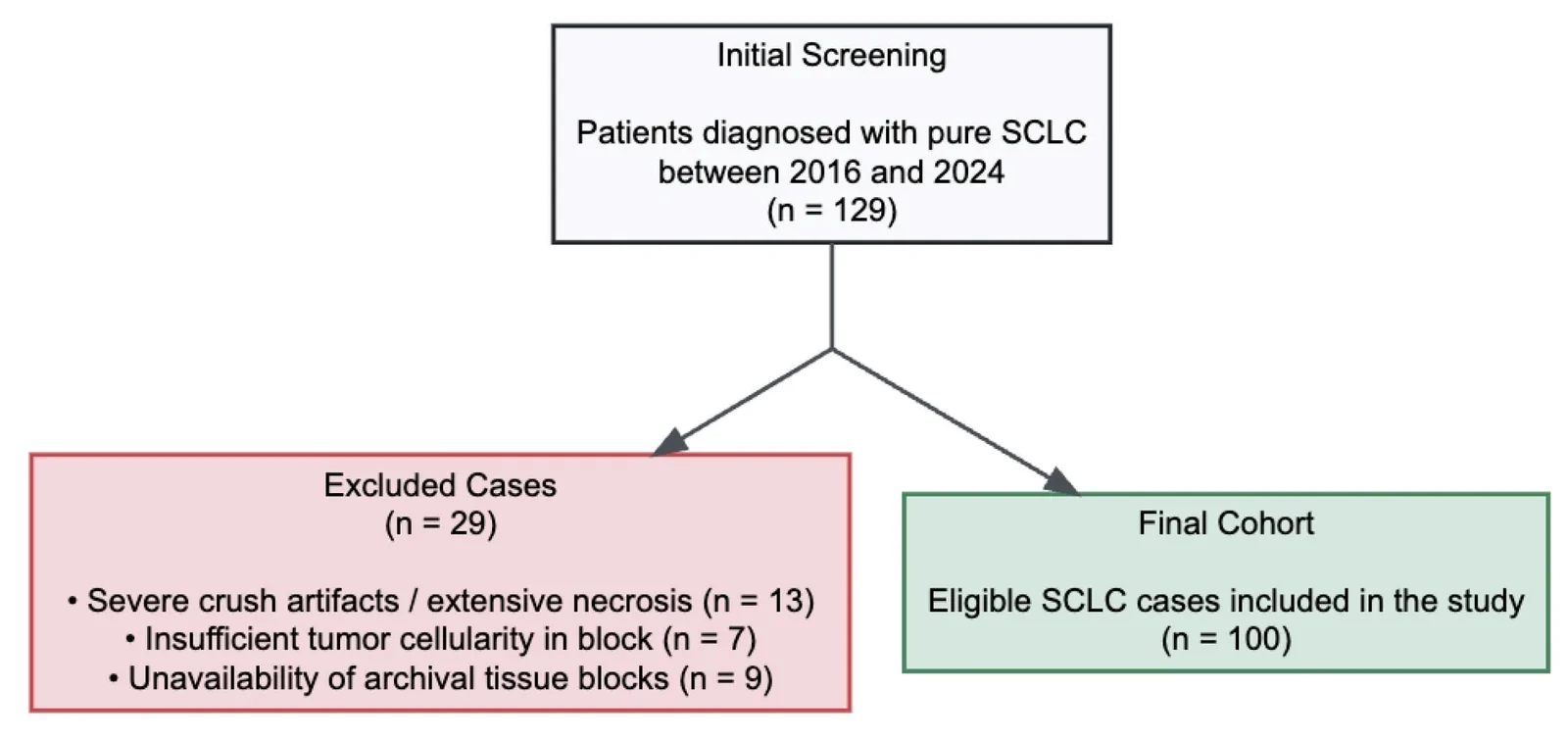
Flow diagram illustrating patient selection, inclusion, and exclusion criteria.

A total of 129 retrospective cases diagnosed with pure SCLC between 2016 and 2024 at the Medical Oncology Department of Çukurova University Faculty of Medicine were initially screened. Hematoxylin and eosin (H&E)-stained sections of all cases were independently re-evaluated by two pathologists according to the updated World Health Organization (WHO) classification criteria for thoracic tumors, with any baseline discrepancies resolved by consensus. Twenty-nine cases were subsequently excluded from the study cohort based on predefined criteria: 13 due to severe crushing artifacts or widespread necrosis rendering the tissue unsuitable for evaluation, 7 due to insufficient viable tumor cellularity remaining in the tissue block, and 9 due to unavailability of the archived block (Figure 1). Consequently, a final cohort of 100 pure SCLC cases met all protocol criteria and was included in the study.

**Figure 2:**
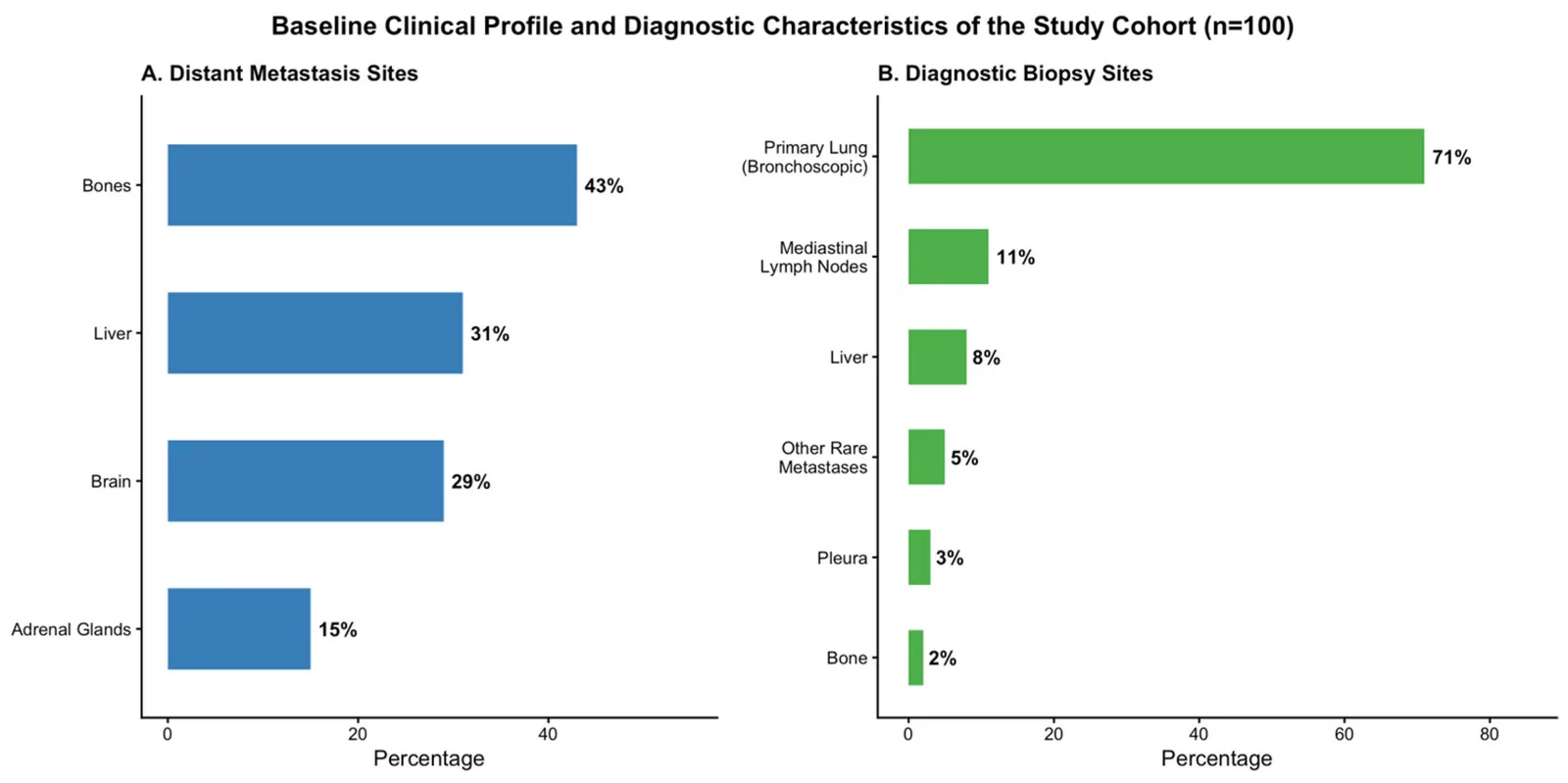
Baseline clinical profile of the study cohort (*n* = 100). (**A**) Prevalence and distribution of specific distant metastatic sites detected at initial staging. (**B**) Anatomical distribution of the primary biopsy foci utilized for histopathological and immunohistochemical confirmation.

Baseline clinicopathological characteristics—including chronological age, sex, smoking status, date of initial diagnosis, diagnostic tissue source (pulmonary parenchymal biopsy, mediastinal lymph node sampling, or distant visceral metastasis), and anatomical tumor stage stratified according to the Veterans Administration Lung Study Group (VALG) criteria (Limited Stage vs. Extensive Stage) (Figure 2)—were systematically extracted from electronic medical records. Additionally, specific synchronous metastatic sites, anatomical disease distribution, precise therapeutic regimens (standard platinum-based doublet chemotherapy vs. first-line chemoimmunotherapy), and long-term survival outcomes were documented. This study protocol was formally approved by the Non-Interventional Clinical Research Ethics Committee of Çukurova University Faculty of Medicine on 14 June 2024 (Meeting No: 145, Decision No: 86) and was conducted in strict compliance with the ethical tenets of the Declaration of Helsinki.

### 2.2. Immunohistochemistry Scoring and Subtype Classification

Formalin-fixed, paraffin-embedded (FFPE) archival blocks from confirmed pure SCLC cases were retrieved, and 5 μm thick serial sections were obtained. Quantitative immunohistochemical (IHC) profiling was performed using a BenchMark XT automated staining platform. Sections were incubated with primary antibodies against NEUROD1 (clone HL3356, GeneTex, Irvine, CA, USA), ASCL1 (polyclonal, Invitrogen, Carlsbad, CA, USA), YAP1 (clone KOLN 63.7, Santa Cruz Biotechnology, Santa Cruz, CA, USA), and POU2F3 (clone E5N2D, Cell Signaling Technology, Danvers, MA, USA). Conventional neuroendocrine markers—including synaptophysin, chromogranin A, CD56, and TTF-1—were concurrently evaluated.

All prepared slides were independently examined by two pathologists at predefined optical magnifications using an Olympus BX53 light microscope (Olympus Corporation, Tokyo, Japan). Any numerical scoring discrepancies were resolved by simultaneous evaluation on a multi-head shared microscope. Human small bowel tissue served as the positive control for NEUROD1, colon mucosa for YAP1, appendix for POU2F3, and a high-grade neuroendocrine tumor for ASCL1. Distinct nuclear localization was defined as specific positivity for ASCL1, NEUROD1, and POU2F3, whereas both clear nuclear and cytoplasmic immunoreactivity were considered positive for YAP1. Expression levels for each lineage driver were quantified as a continuous variable by calculating the exact percentage of positive-staining viable tumor cells across the entire slide area.

To ensure translational reproducibility, asymmetric immunohistochemical expression cut-offs were strictly adopted from the landmark multi-center reference study by Megyesfalvi et al. [16]: ≥50% for ASCL1, ≥5% for NEUROD1, ≥1% for POU2F3, and >0% for YAP1. These established cut-offs have been validated to tightly correlate IHC staining at the proteomic level with underlying RNA transcriptomic profiles [16]. Tumors expressing only a single lineage driver above these thresholds were classified as the corresponding mono-dominant molecular subtype (SCLC-A, SCLC-N, or SCLC-P). Tumors displaying concurrent marker expression exceeding these thresholds were designated as co-dominant hybrid phenotypes, including SCLC-AN (ASCL1/NEUROD1) and SCLC-AP (ASCL1/POU2F3) [17]. Tumors lacking immunoreactivity for all four lineage markers were classified as quadruple-negative SCLC. Within this parsimonious architectural framework, YAP1 expression dynamics were independently quantified to characterize its distribution across the cohort.

### 2.3. Chemoimmunotherapy Subcohort and Clinical Efficacy Assessment

To investigate the potentially predictive clinical value of immunohistochemical lineage subtyping, an exploratory analysis was conducted on a subcohort (*n* = 19) of patients with extensive-stage disease who received standard first-line platinum-based doublet chemotherapy concurrently with an anti-PD-L1 immune checkpoint inhibitor (specifically atezolizumab). Due to sample size constraints within individual subclonal lines, patients were dichotomized into two analytical cohorts based on neuroendocrine marker accumulation: the NE-high group (*n* = 16), comprising the SCLC-A and SCLC-AN phenotypes, and the NE-low/Non-NE group (*n* = 3), comprising the SCLC-P, SCLC-AP, and SCLC-QN phenotypes. Within this exploratory framework, short-term therapeutic efficacy was evaluated using the objective response rate (ORR), defined as the percentage of patients achieving a complete or partial response in strict accordance with the Response Evaluation Criteria in Solid Tumors (RECIST) version 1.1. Long-term clinical survival endpoints across these immunophenotypic trajectories were subsequently quantified using overall survival (OS) analyses.

### 2.4. Statistical Analysis

Statistical analyses were conducted using Jamovi (version 2.6.44). Advanced data visualizations, risk-stratified Kaplan–Meier survival curves, multivariate forest plots, and expression heatmaps were generated within the RStudio (RStudio version 2024.04.2+764 (or aligned R version 4.4.1)) environment utilizing the *ggplot2*, *survival*, *survminer*, *cowplot*, *forestplot*, and *pheatmap* packages. Categorical variables were summarized as frequencies (*n*) and percentages (%). Continuous variables were assessed for normality using the Shapiro–Wilk test. Normally distributed continuous parameters were reported as mean ± standard deviation (SD), whereas skewed quantitative proteomic percentages were summarized using median and interquartile range (IQR). Group means for continuous age dynamics were compared using one-way ANOVA.

### 2.5. Group Comparisons

Categorical distributions across SCLC molecular subtypes were evaluated using the Pearson chi-square (χ^2^) test or Fisher’s exact test (utilizing Monte Carlo simulation parameters with 2000 replicates where necessary) when expected cell frequencies were less than 5 to maintain statistical integrity within limited subclonal cohorts. Following significant chi-square distributions, standardized residuals were examined as a post hoc evaluation to isolate significant cell-specific deviations, where absolute values greater than 2.0 (|residual| > 2.0) indicated significant cell divergence from the null expected value. Comparisons of continuous quantitative biomarker expression percentages among subtypes were performed using the nonparametric Kruskal–Wallis test, followed by post hoc pairwise evaluations using the Dwass–Steel–Critchlow–Fligner (DSCF) method. For all baseline statistical evaluations, the significance threshold was set at *p* < 0.05, and confidence intervals were calculated at the 95% level.

### 2.6. Survival Analyses and Multivariate Modeling Architecture

Overall survival (OS) was defined as the time from initial histopathological diagnosis to the date of death or the last documented clinical follow-up. Longitudinal survival trajectories were estimated using the Kaplan–Meier method, and differences between stratified phenotypic cohorts were evaluated using the two-tailed log-rank test. Median survival times and corresponding 12-, 36-, and 60-month survival probabilities were reported with their 95% confidence intervals (CIs). Univariate and multivariate Cox proportional hazards regression models were used to evaluate the baseline prognostic effects of clinical and molecular indicators on overall survival clinical endpoints.

To avoid statistical overfitting and unstable risk coefficient estimates driven by low event-to-variable ratios, a parsimonious multivariate Cox regression architecture was adopted. Covariate entry options within the multivariate matrix were strictly limited to robust traditional staging dimensions (Limited Stage vs. Extensive Stage), synchronous bone metastasis profiles, first-line systemic therapeutic interventions (chemoimmunotherapy vs. chemotherapy alone) as an operational baseline covariate, and quantitative continuous ASCL1 and NEUROD1 expression percentages. Rare subclonal cells with extreme sample size constraints (*n* = 1 or *n* = 2) were excluded from multivariate matrix modeling to maintain coefficient stability. The proportional hazards assumption across all Cox regression architectures was verified using Schoenfeld residual diagnostics, confirming that the assumption was not violated (*p* > 0.05). All statistical tests were two-tailed, and *p* < 0.05 was considered statistically significant.

For the exploratory therapeutic subgroup (*n* = 19) receiving first-line platinum–etoposide concurrently with atezolizumab, Fisher’s exact test was used to compare objective tumor response rates (ORR; Responders [complete/partial response] vs. Non-responders [stable/progressive disease]) between the neuroendocrine burden groups (NE-high vs. NE-low/Non-NE) due to small cell sample size limitations. Longitudinal survival timelines between these immunophenotypic cohorts were estimated using Kaplan–Meier, and the prognostic divergence between the risk trajectories was assessed using a two-tailed log-rank framework.

## 3. Results

### 3.1. Baseline Clinicopathological Characteristics

The baseline clinicopathological characteristics of the complete study cohort (*n* = 100) are summarized in Supplementary Table S1. The mean chronological age at initial diagnosis was 62.1 ± 8.7 years (range: 33–78 years). The study population showed a significant male predominance, with 77% (*n* = 77) male and 23% (*n* = 23) female patients. A documented history of tobacco smoking was present in 68% (*n* = 68) of cases, and at least one baseline clinical comorbidity was documented in 52% (*n* = 52) of patients. At initial clinical staging performed in accordance with the Veterans Administration Lung Study Group (VALG) criteria, 31% (*n* = 31) of patients presented with limited-stage disease (LS-SCLC), whereas the remaining 69% (*n* = 69) presented with extensive-stage disease (ES-SCLC).

Among the entire population, the most frequent sites of synchronous distant metastatic involvement at baseline staging were the bones (43%), followed by the liver (31%), brain (29%), and adrenal glands (15%) (Figure 2A). For histopathological and immunohistochemical confirmation, the primary diagnostic biopsy foci were most often the primary lung tumor sampled via bronchoscopic interventions (71%). This was followed by mediastinal lymph nodes (11%), liver lesions (8%), pleural tissue (3%), and bone lesions (2%). In the remaining 5% of cases, histopathological and immunohistochemical confirmation was obtained from other less common metastatic foci (Figure 2B).

### 3.2. Immunohistochemical Expression Profiles of Master Transcription Factors

**Figure 3:**
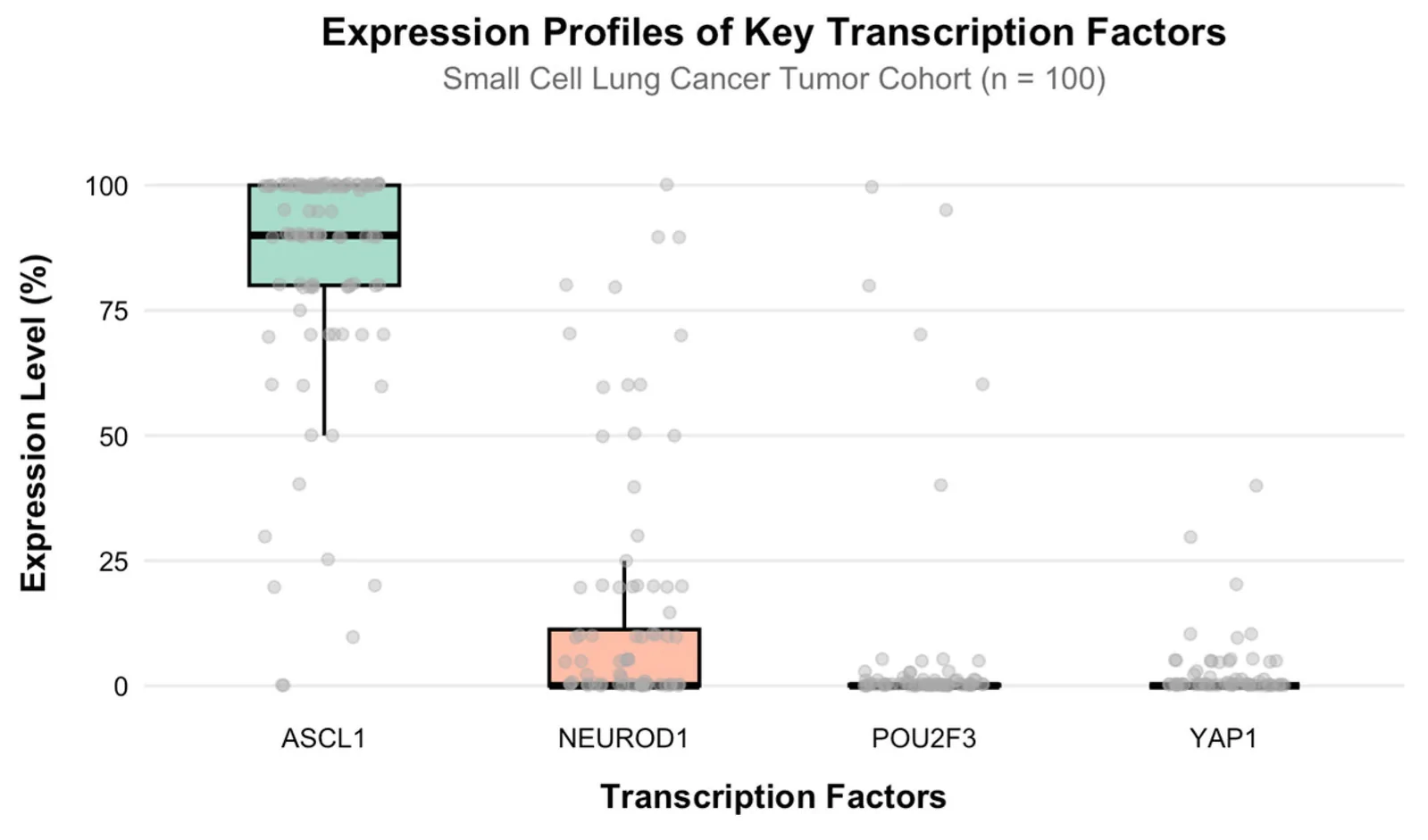
Quantitative distribution profiles of master lineage transcription factors across the SCLC cohort (*n* = 100). Box plots illustrate the percentage distributions of immunoreactive tumor cells for ASCL1, NEUROD1, POU2F3, and YAP1. Horizontal lines within the boxes designate median values, box boundaries define the interquartile ranges, and individual gray dots represent single clinical tumor specimens.

**Figure 4:**
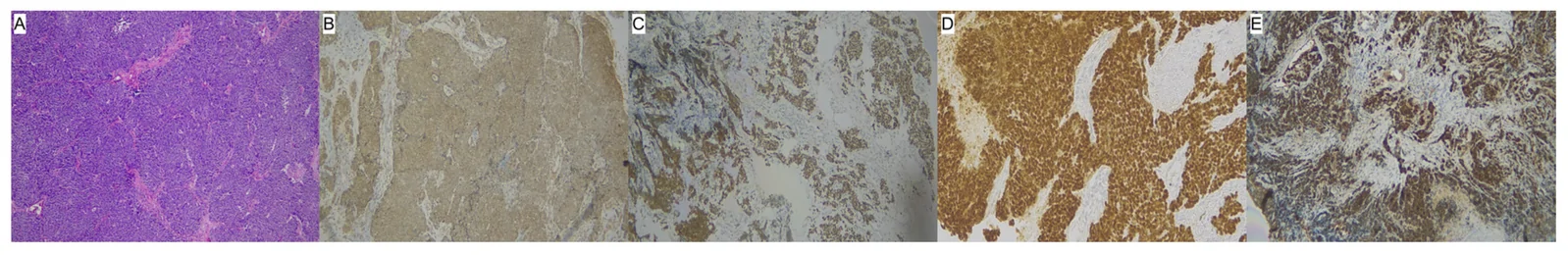
Representative histopathological features and lineage-specific immunohistochemical (IHC) expression patterns in pure small-cell lung cancer. (**A**) Standard Hematoxylin and Eosin (H&E) staining showing small-cell lung carcinoma sheets characterized by scant cytoplasm, granular nuclear chromatin, and characteristic crushing artifacts. Serial tissue sections demonstrate the distinct brown subcellular localization of key transcription factors, including diffuse nuclear expression for ASCL1 (**B**), NEUROD1 (**C**), and POU2F3 (**D**), along with both nuclear and cytoplasmic immunoreactivity for YAP1 (**E**) (Original magnification: ×200 and ×400).

When the immunoreactivity profiles of the four master lineage transcription factors were quantified within the malignant epithelial population across the entire study cohort (*n* = 100), ASCL1 emerged as the biomarker with the highest baseline prevalence. ASCL1 showed positive expression above the validation threshold in 97% (*n* = 97) of SCLC cases, with a median expression of 90.0% (interquartile range [IQR]: 20.0%). NEUROD1 exhibited positive immunoreactivity in 45% (*n* = 45) of patients, with a median of 0.0% and an IQR of 11.2%. Furthermore, POU2F3 expression was detected above the established positivity cut-off in 25% (*n* = 25) of cases (median: 0.0%, IQR: 0.2%), whereas YAP1 positivity was documented in 24% (*n* = 24) of the cohort (median: 0.0%, IQR: 0.0%) (Figure 3). The precise tissue architectures, cytomorphological variants, and respective subcellular localization profiles (nuclear versus cyto-nuclear immunoreactivity) are illustrated in representative tissue sections (Figure 4).

### 3.3. Inter-Marker Correlation Matrix Analysis

**Figure 5:**
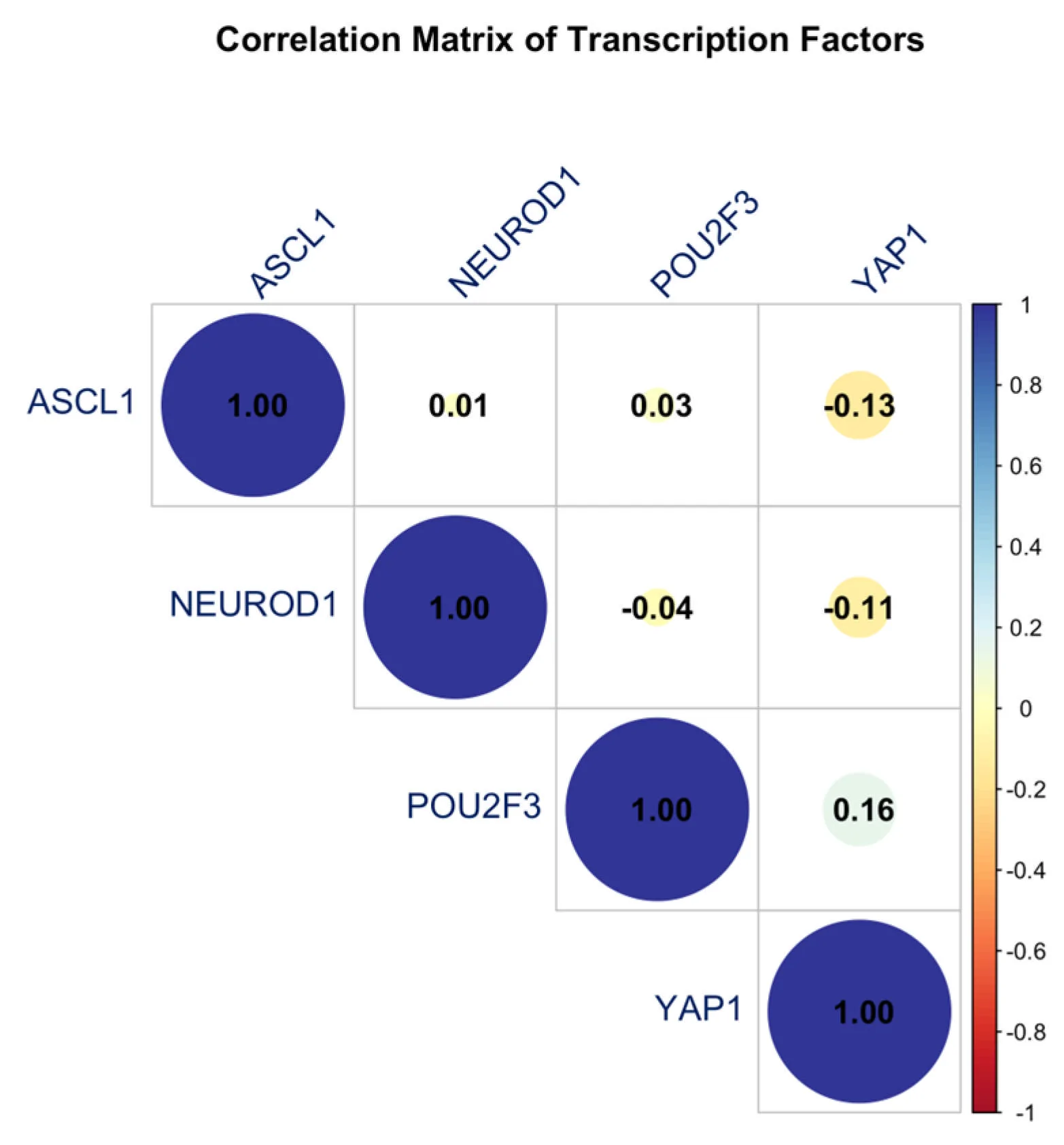
Correlation matrix of master transcription factor expression levels. Circle sizes and corresponding numbers represent individual Spearman’s rank correlation coefficients (ρ) among continuous ASCL1, NEUROD1, POU2F3, and YAP1 tumor percentages. The scale on the right defines the correlation strength, ranging from −1.00 (red, negative correlation) to +1.00 (blue, positive correlation).

To evaluate potential biological interactions and co-expression dynamics among the quantitative expression levels of ASCL1, NEUROD1, POU2F3, and YAP1, a pairwise Spearman’s rank correlation analysis was performed. No strong correlations were observed among the four transcription factors, with all correlation coefficients (ρ) clustering close to zero. A weak positive correlation was observed exclusively between POU2F3 and YAP1 expressions (ρ = 0.16), whereas a minor negative correlation trend was documented between ASCL1 and YAP1 (ρ = −0.13). The remaining pairwise continuous evaluations demonstrated independent transcriptional behaviors across samples: ASCL1–NEUROD1 (ρ = 0.01), ASCL1–POU2F3 (ρ = 0.03), NEUROD1–POU2F3 (ρ = −0.04), and NEUROD1–YAP1 (ρ = −0.11) (Figure 5).

### 3.4. Molecular Subtype Distributions

**Figure 6:**
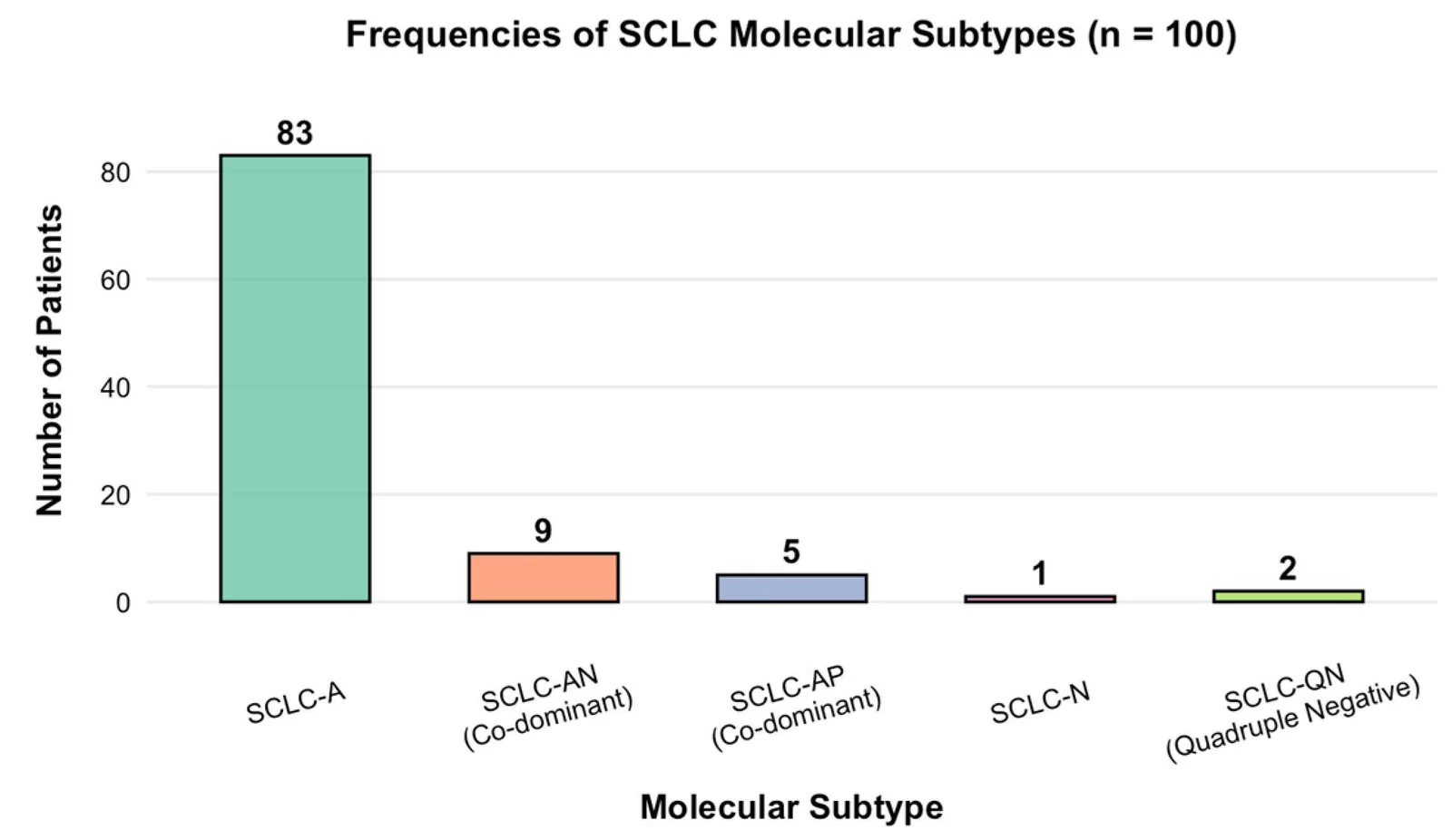
Percentage and frequency distribution breakdown of IHC-defined SCLC molecular subtypes. The chart illustrates the stratification into mono-dominant (SCLC-A, SCLC-N), hybrid/co-dominant (SCLC-AN, SCLC-AP), and quadruple-negative (SCLC-QN) phenotypic subsets within the study cohort (*n* = 100). SCLC, small-cell lung cancer; IHC, immunohistochemistry.

**Figure 7:**
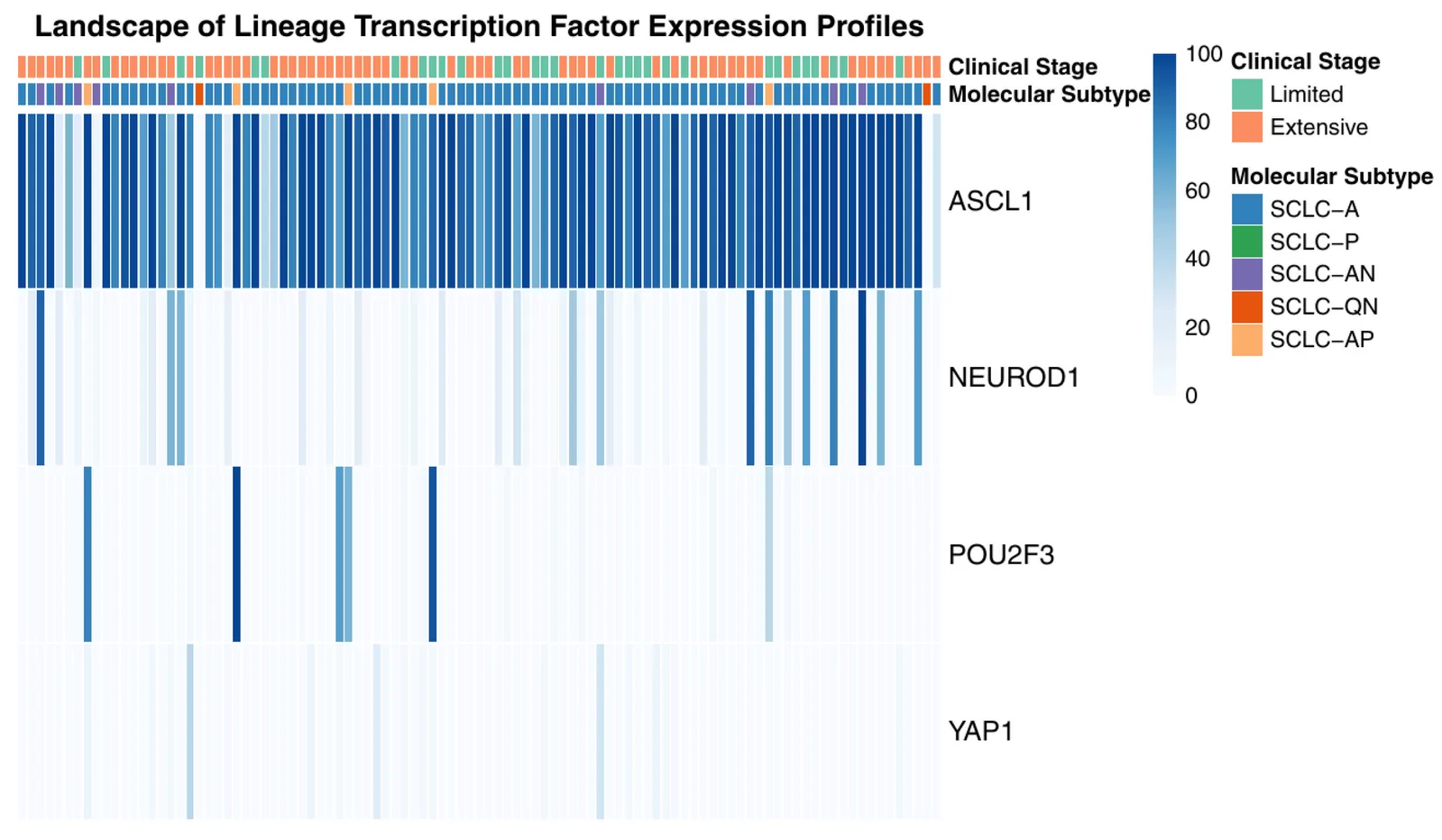
Clinicopathological landscape and immunohistochemical (IHC) expression profiles of master transcription factors across the study cohort (*n* = 100). Individual columns represent single patient samples, and rows indicate ASCL1, NEUROD1, POU2F3, and YAP1 expression percentages. The color scale indicates IHC expression intensities, ranging from light blue/white (0%, low/negative) to deep blue (100%, high). Layered color bars at the top indicate molecular subtype assignments and baseline Veterans Administration Lung Study Group (VALG) clinical stages. SCLC-N patients are excluded to enhance visual clarity and maintain balance. SCLC, small-cell lung cancer.

Using the predefined asymmetric multi-marker positivity thresholds on the immunophenotypic profiles, the final distribution of the 100 pure SCLC cases across molecular subsets was determined. The single-dominant SCLC-A subtype accounted for the majority of the cohort, comprising 83% (*n* = 83) of cases. Among cases with co-expression of multiple master transcription factors above the clinical cut-offs, 9% (*n* = 9) were stratified into the co-dominant SCLC-AN category, and 5% (*n* = 5) were classified as the co-dominant SCLC-AP subtype. Conversely, the single-dominant SCLC-N phenotype was highly restricted, occurring in only 1% (*n* = 1) of the study population. Finally, tumors completely lacking all master transcription factors were categorized as SCLC-QN, representing 2% (*n* = 2) of the entire cohort (Figure 6 and Figure 7).

### 3.5. Subtype-Specific Expression Dynamics of Master Transcription Factors

Cross-tabulation analyses revealed statistically significant differences among the IHC-defined SCLC molecular subtypes in both categorical positivity rates and continuous quantitative expression levels for ASCL1 (*p* < 0.001), NEUROD1 (*p* = 0.001), and POU2F3 (*p* = 0.002) (Table 1). From a continuous quantitative perspective, post hoc pairwise comparisons showed that NEUROD1 expression levels were significantly higher in the co-dominant SCLC-AN subgroup than in the mono-dominant SCLC-A subset (*p* < 0.001). Similarly, POU2F3 expression levels were significantly higher in the co-dominant SCLC-AP cohort than in both the SCLC-A (*p* < 0.001) and SCLC-AN (*p* = 0.008) phenotypic groups. Conversely, baseline evaluations showed no statistically significant differences across the molecular variants in either categorical YAP1 positivity or continuous quantitative YAP1 expression dynamics (*p* = 0.695), reflecting a uniform distribution of this marker across the cohort.

### 3.6. Correlations with Conventional Pathological and Neuroendocrine Biomarkers

When examining correlations with traditional thoracic pathological markers, TTF-1 expression showed a borderline association with molecular subtype (*p* = 0.054). In contrast, total cumulative neuroendocrine marker scores (comprising synaptophysin, chromogranin A, and CD56 co-expression metrics) were uniform across all phenotypic subsets, with a positive rate of 96.3% (*p* = 0.962). Although categorical CD56 positivity showed statistical differences across subsets (*p* = 0.002), this sub-evaluation had missing archival data; thus, this finding should be interpreted with caution. Detailed subgroup test statistics, exact chi-square (χ^2^) values, post hoc standardized residuals, and pairwise continuous comparison matrices are provided in Supplementary Table S2.

### 3.7. Relationship Between Molecular Subtypes, Clinicopathological Features, and Patterns of Distant Metastasis

Cross-tabulation analyses revealed no statistically significant associations between the IHC-defined SCLC molecular subtypes and baseline demographic or clinical characteristics, including age at diagnosis (*p* = 0.974), sex (*p* = 0.560), clinical comorbidities (*p* = 0.597), and smoking history (*p* = 0.247) (Table 1). Disease stage at initial presentation (limited-stage versus extensive-stage SCLC) did not significantly correlate with the transcription factor-driven molecular classification (*p* = 0.905). No evidence of subtype-specific clustering by disease stage was observed across the entire cohort or within the distinct transcription factor expression profiles (Figure 7).

Similarly, baseline distant metastases showed a high degree of uniformity across the cohort and did not differ significantly among the stratified molecular subgroups. This included individual rates for liver metastasis (*p* = 0.498), brain metastasis detected at baseline (*p* = 0.371), bone metastasis (*p* = 0.461), and adrenal gland involvement (*p* = 0.782). Furthermore, longitudinal tracking revealed no significant difference between the cohorts in the subsequent development of brain metastasis during the disease course (*p* = 0.311). Finally, there was no statistically significant divergence in disease recurrence rates among limited-stage SCLC patients following primary therapy across subtypes (*p* > 0.05) (Table 1).

### 3.8. Univariate Survival Profiling of Master Transcription Factors

**Figure 8:**
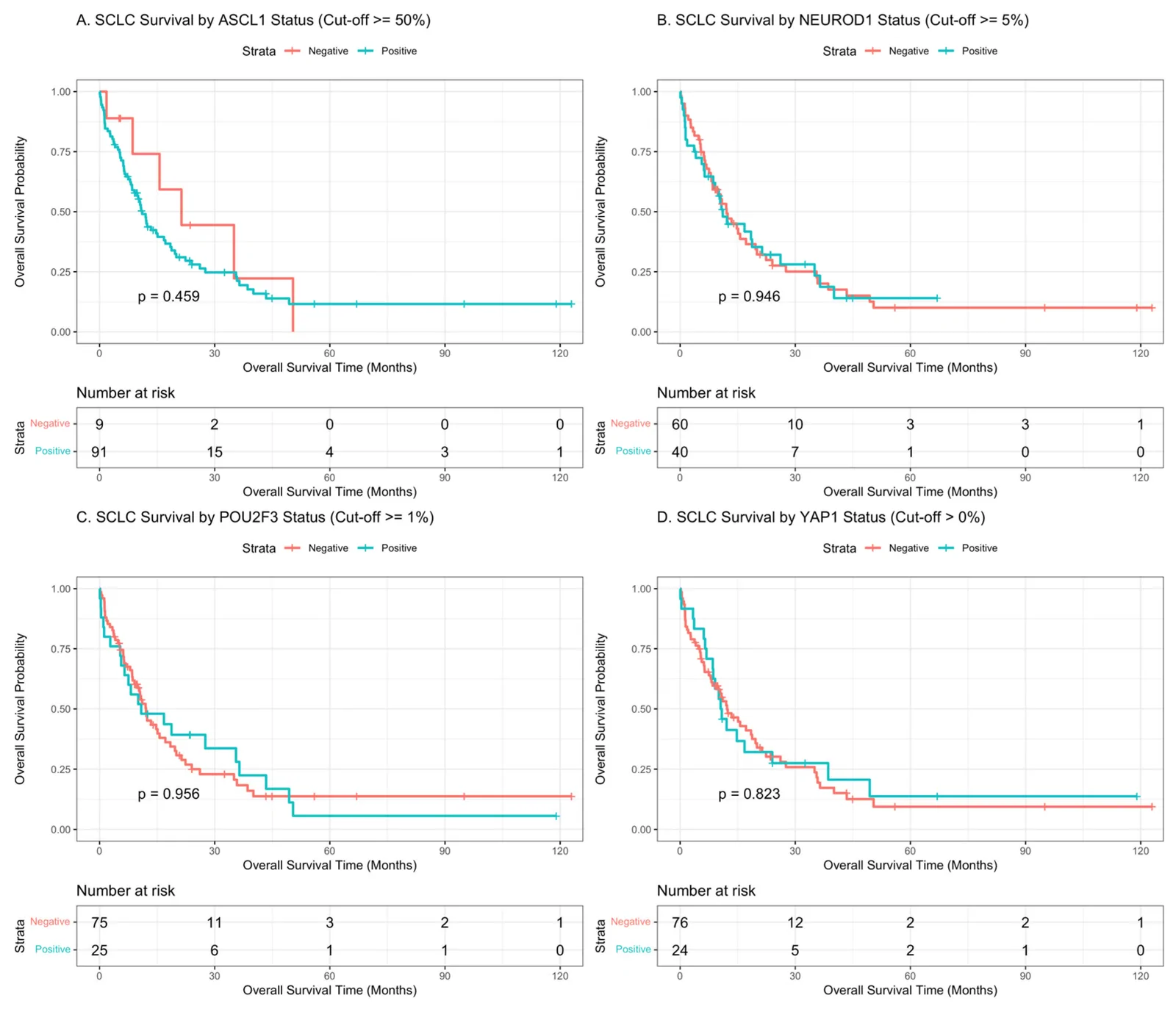
Kaplan–Meier overall survival curves stratified by lineage transcription factor expression profiles in the SCLC cohort (*n* = 100). Patients are dichotomized using predefined immunohistochemical cut-off thresholds for each master regulator. (**A**) Overall survival stratified by ASCL1 status (cut-off ≥ 50%), displaying a lack of statistically significant survival variance between the cohorts (*p* = 0.459). (**B**) Overall survival stratified by NEUROD1 status (cut-off ≥ 5%), demonstrating overlapping survival trajectories (*p* = 0.946). (**C**) Overall survival stratified by POU2F3 status (cut-off ≥ 1%), displaying similar survival outcomes (*p* = 0.956). (**D**) Overall survival stratified by YAP1 status (cut-off > 0%), displaying no significant survival variations across groups (*p* = 0.823).

The baseline prognostic impacts of the four master lineage transcription factors on overall survival (OS) clinical endpoints within the study cohort (*n* = 100) were evaluated using univariate Cox proportional hazards regression and Kaplan–Meier log-rank analyses (Table 2). Within the cohort characterized by ASCL1 negativity (*n* = 9), the median OS was 21.4 months (95% confidence interval [CI]: 8.6–not applicable [NA]), whereas patients presenting with ASCL1 positivity (*n* = 91) displayed a median OS of 11.1 months (95% CI: 8.5–17.1 months) (Table 2). The estimated 12-month survival rates were 74.1% (95% CI: 48.4–100.0%) for the ASCL1-negative group and 47.7% (95% CI: 38.2–59.5%) for the ASCL1-positive cohort. Although a visual survival separation favoring the ASCL1-negative group was documented, the univariate Cox hazard ratio (HR) for the ASCL1-positive subset was 1.37 (95% CI: 0.59–3.16), demonstrating no statistically significant prognostic association (*p* = 0.459; Figure 8A).

The median OS for the NEUROD1-negative (*n* = 60) and NEUROD1-positive (*n* = 40) patient cohorts was 12.1 months (95% CI: 8.4–20.0 months) and 11.1 months (95% CI: 8.6–26.1 months), respectively (Table 2). Univariate proportional hazards regression demonstrated overlapping survival trajectories, yielding an HR of 1.02 (95% CI: 0.64–1.62; *p* = 0.946; Figure 8B). For patients stratified by POU2F3 expression, the median OS durations were 12.1 months (95% CI: 9.8–18.5 months) for the negative group (*n* = 75) and 10.8 months (95% CI: 6.5–43.4 months) for the positive group (*n* = 25) (Table 2). The unadjusted HR for POU2F3 positivity was 0.99 (95% CI: 0.59–1.64), indicating statistically similar survival outcomes (*p* = 0.956; Figure 8C). Finally, evaluations demonstrated a median OS of 12.2 months (95% CI: 8.4–19.6 months) for the YAP1-negative group (*n* = 76) and 10.7 months (95% CI: 8.6–38.6 months) for the YAP1-positive group (*n* = 24) (Table 2). The univariate HR for the YAP1-positive cohort was 0.94 (95% CI: 0.56–1.59), confirming that this marker displayed no statistically significant impact on unadjusted baseline survival endpoints (*p* = 0.823; Figure 8D).

### 3.9. Multivariate Survival Modeling and Independent Prognostic Predictors

**Figure 9:**
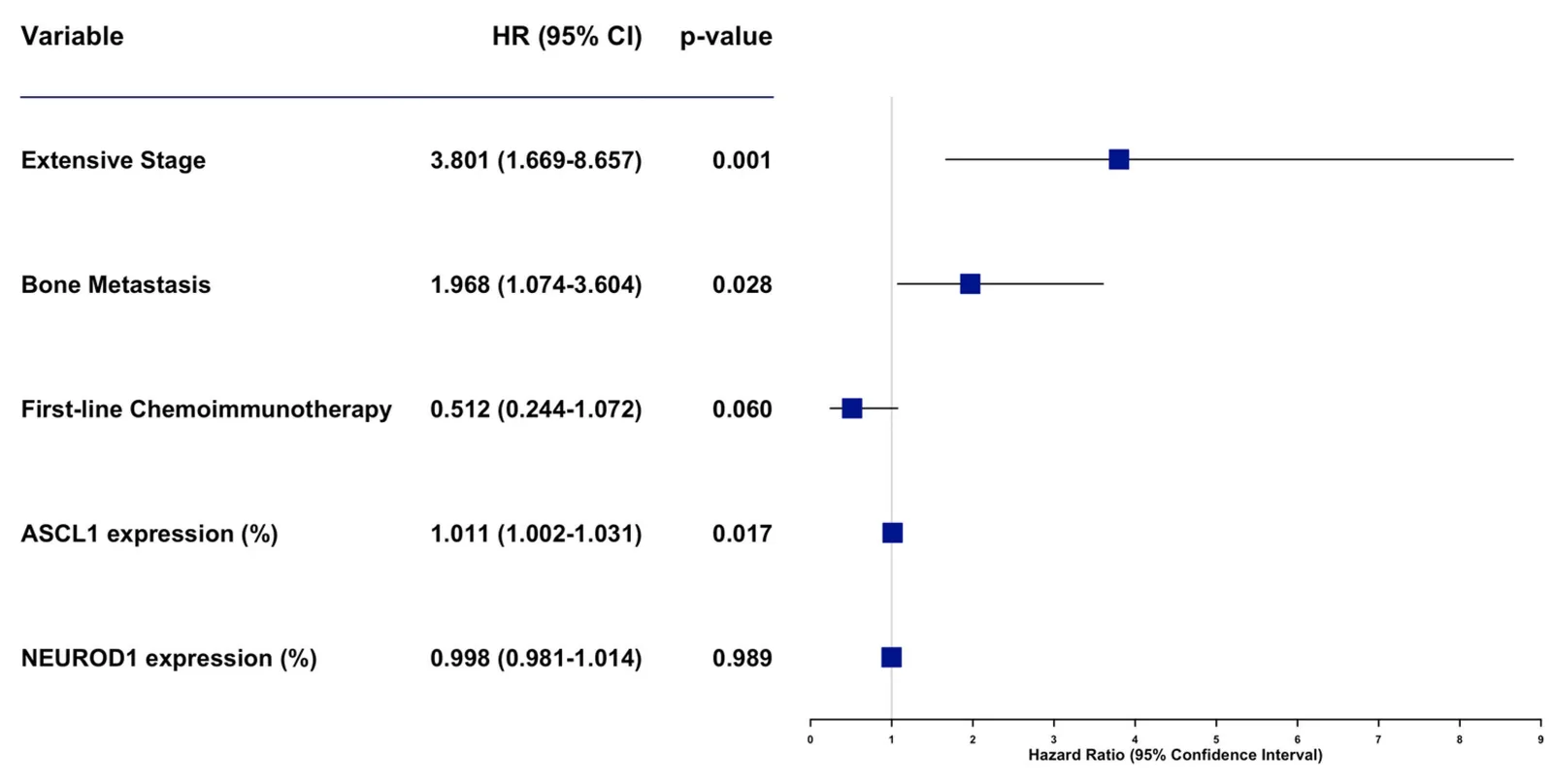
Forest plot illustrating the treatment-adjusted parsimonious multivariate Cox proportional hazards regression analysis of independent predictors of overall survival. Hazard ratios (HR) are represented as solid squares, with horizontal lines defining the corresponding 95% confidence intervals (CI). The vertical solid reference line at 1.00 indicates the null hypothesis. Extensive stage disease (HR = 3.801, 95% CI: 1.669–8.657, *p* = 0.001) and synchronous bone metastasis at presentation (HR = 1.968, 95% CI: 1.074–3.604, *p* = 0.028) represent the statistically significant independent clinical predictors of worse outcomes. Continuous baseline ASCL1 percentage demonstrates a minor independent prognostic association (HR = 1.011, 95% CI: 1.002–1.031, *p* = 0.017), whereas first-line chemoimmunotherapy exhibits a protective numerical trend (HR = 0.512, 95% CI: 0.244–1.072, *p* = 0.060). SCLC, small-cell lung cancer.

To address statistical overfitting driven by low event-to-variable ratios and to adjust for confounding clinical covariates across the complete cohort, a treatment-adjusted, parsimonious multivariate Cox proportional hazards regression model was constructed. The hazard ratios and precise risk allocations for these independent prognostic indicators are shown in the multivariate forest plot (Figure 9). After statistical adjustment for first-line systemic treatment modalities (chemoimmunotherapy vs. chemotherapy alone) as a baseline clinical covariate, extensive-stage disease emerged as the strongest independent predictor of poor survival, yielding a 3.801-fold higher risk of death (HR = 3.801, 95% CI: 1.669–8.657, *p* = 0.001; Figure 9).

The clinical presence of synchronous bone metastases at initial presentation was also confirmed as an independent predictor of worse clinical outcomes, with a nearly two-fold increase in risk (HR = 1.968, 95% CI: 1.074–3.604, *p* = 0.028; Figure 9). Notably, within this treatment-adjusted, parsimonious matrix, first-line chemoimmunotherapy showed a protective numerical trend, reducing the risk of death by 49% compared with chemotherapy alone, although it did not reach conventional statistical significance thresholds within this unselected cohort (HR = 0.512, 95% CI: 0.244–1.072, *p* = 0.060; Figure 9).

Quantitative, continuous multivariate tracking unmasked a minor but statistically independent prognostic association for baseline continuous ASCL1 percentage (HR = 1.011, 95% CI: 1.002–1.031, *p* = 0.017), indicating that, after adjusting for baseline clinical parameters, each unit increase in continuous ASCL1 expression contributes linearly to worse survival outcomes. Conversely, continuous NEUROD1 percentage showed no prognostic relevance for overall survival endpoints within the adjusted multivariate matrix (HR = 0.998, 95% CI: 0.981–1.014, *p* = 0.989; Figure 9). Other baseline covariates—including demographic characteristics, liver or brain metastases, and discrete multi-marker molecular sub-stratifications—showed no independent prognostic associations with long-term survival clinical endpoints within the adjusted parsimonious framework (all *p* > 0.05).

### 3.10. Exploratory Efficacy and Survival Analysis of the First-Line Chemoimmunotherapy Subcohort

An exploratory analysis was performed on a subcohort of 19 SCLC patients with extensive-stage disease who received initial platinum–etoposide chemotherapy combined with an anti-PD-L1 immune checkpoint inhibitor to evaluate how immunohistochemical lineage subtyping correlates with clinical treatment outcomes. All patients in this subcohort received systemic treatment with atezolizumab. Due to sample size limitations within individual lines, patients were dichotomized into two categories based on neuroendocrine marker baseline accumulation: the NE-high group, which includes mono-dominant SCLC-A and co-dominant SCLC-AN phenotypes (*n* = 16), and the NE-low/Non-NE group, consisting of SCLC-P, SCLC-AP, and SCLC-QN phenotypes (*n* = 3).

**Figure 10:**
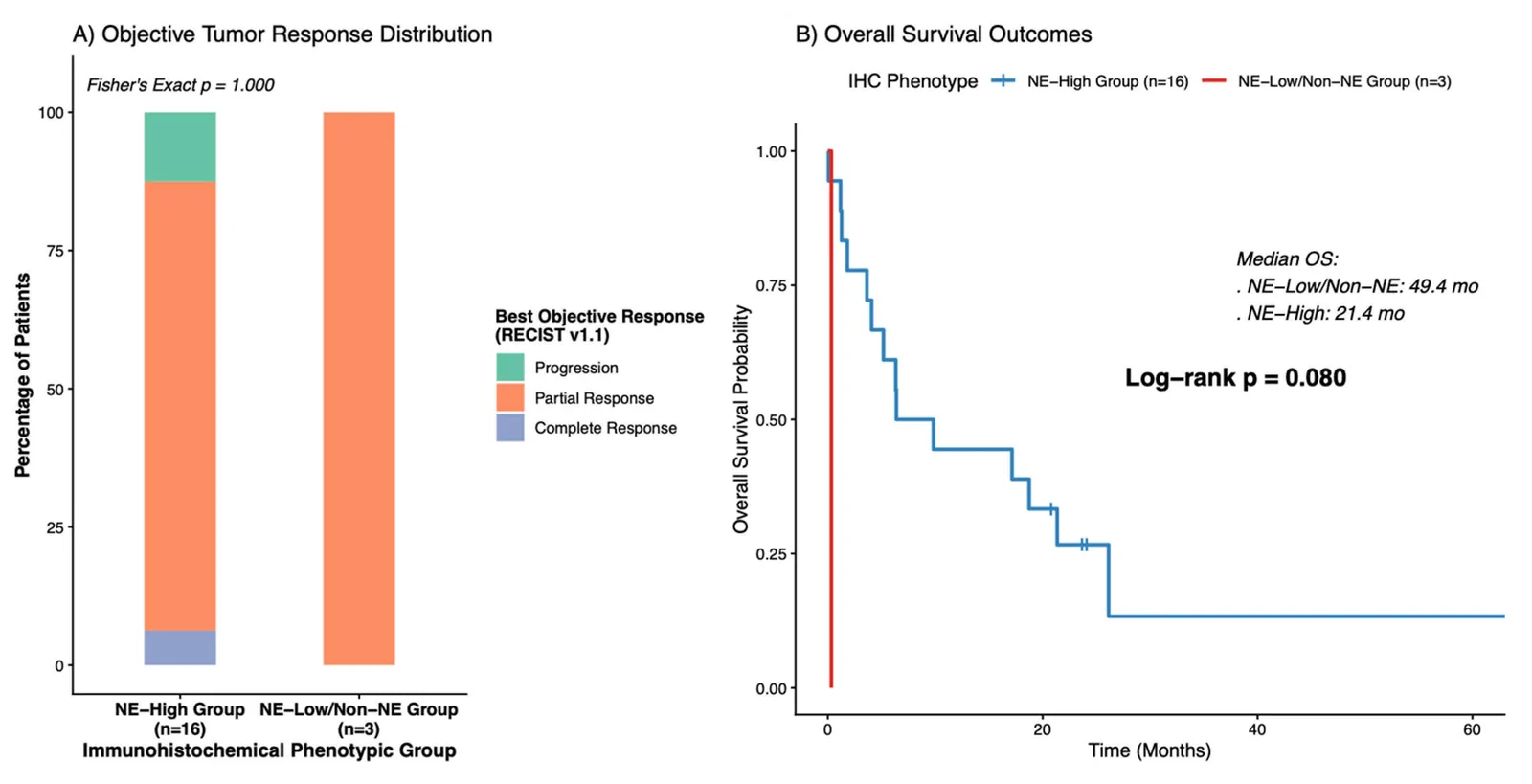
Exploratory therapeutic efficacy and overall survival outcomes in the first-line chemoimmunotherapy subcohort (Atezolizumab, *n* = 19). (**A**) Stacked bar chart showing the distribution of best objective tumor responses assessed using the RECIST version 1.1 parameters. The NE-low/Non-NE group achieved an objective response rate (ORR) of 100.0% (*n* = 3/3), characterized by sustained partial responses (PR). The NE-high group demonstrated an ORR of 87.5% (*n* = 14/16), including 1 complete response (CR), 13 partial responses (PR), and 2 cases of progressive disease (PD) (Fisher’s exact test, *p* = 1.000). (**B**) Kaplan–Meier overall survival curves stratified by the immunohistochemical (IHC)-based phenotypic groups. Patients within the NE-low/Non-NE group (*n* = 3; red line) demonstrated a median overall survival of 49.4 months, compared with 21.4 months for those within the NE-high group (*n* = 16; blue line) (two-tailed log-rank test, *p* = 0.080). SCLC, small-cell lung cancer; RECIST, Response Evaluation Criteria in Solid Tumors.

Regarding short-term therapeutic efficacy, the best objective tumor response was quantified using the RECIST version 1.1 criteria. Within the NE-high group, the objective response rate (ORR) was 87.5% (*n* = 14/16), comprising a partial response (PR) in 13 patients, a confirmed complete response (CR) in 1 patient, and progressive disease (PD) in 2 non-responding cases. Conversely, all 3 patients in the NE-low/Non-NE group achieved a partial response, yielding an ORR of 100.0% (*n* = 3/3) (Figure 10A). While Fisher’s exact test did not demonstrate statistical significance in response distributions (*p* = 1.000) due to small sample sizes, a clinical response trend was documented within the non-classical phenotypic group.

Longitudinal clinical monitoring revealed a numerical divergence in survival outcomes between the two immunophenotypic cohorts. According to Kaplan–Meier estimates, the median overall survival (mOS) was 21.4 months for the NE-high group, whereas a numerically longer median overall survival of 49.4 months was documented for the NE-low/Non-NE cohort (Figure 10B). Although the conventional significance threshold was not met due to statistical power constraints imposed by the small sample size, log-rank analysis revealed a non-significant numerical trend favoring the non-classical immunophenotypic group between the two survival curves (χ^2^ = 3.0, df = 1, *p* = 0.080; Figure 10B).

## 4. Discussion

This pilot investigation demonstrates the proteomic molecular heterogeneity of SCLC, a disease that has historically been evaluated as a uniform entity managed with standardized chemotherapy regimens based on lineage transcription factors including ASCL1, NEUROD1, POU2F3, and YAP1 [8]. The primary objective of this evaluation was to investigate the feasibility of characterizing these transcriptional profiles—originally defined via RNA sequencing platforms—utilizing quantitative immunohistochemistry (IHC), a pragmatic and cost-effective methodology in pathology workflows [16]. The baseline analysis demonstrated that the four primary transcriptional indicators exhibit a distinct lack of strong linear correlations, with all pairwise Spearman correlation coefficients clustering close to zero (Figure 5). This suggests that these factors operate as independent transcriptional regulators, reflecting subclonal molecular heterogeneity within individual tumor tissues [19]. Within this cohort, ASCL1 was the most prevalent transcription factor (97%), with 83% of patients stratified into the mono-dominant SCLC-A subtype. Furthermore, the hybrid phenotypes SCLC-AN (9%) and SCLC-AP (5%) were documented as co-dominant subsets. YAP1 expression was documented in 24% of the cohort, did not differ significantly across phenotypes (*p* = 0.695), and did not emerge as an independent lineage subtype driver. Finally, clinicopathological assessments revealed no statistically significant associations between these IHC-defined molecular variants and patient baseline demographic characteristics, clinical stage at presentation, or specific anatomical patterns of distant metastasis.

The baseline molecular subtype distribution within this cohort was documented as SCLC-A (83%), SCLC-AN (9%), SCLC-AP (5%), SCLC-QN (2%), and SCLC-N (1%). SCLC-A, characterized by ASCL1 dominance, accounts for approximately 40–70% of SCLC cases across published datasets and remains the most prevalent immunophenotypic variant [7,8]. The current evaluation confirms this dominance, demonstrating an ASCL1 detection rate of 83%. Literature models indicate that ASCL1 operates as a key factor in SCLC oncogenesis, with the tumor mass initially displaying an ASCL1-dominant architecture [8]. However, emerging evidence suggests that SCLC specimens exhibit a homogeneous transcriptomic architecture during the pre-treatment period, whereas lineage plasticity and subclonal transitions are frequently induced following systemic chemotherapy exposure or longitudinal clonal selection [20]. The high prevalence of SCLC-A (83%) documented in the current study may be related to the fact that all evaluated tumor specimens were derived exclusively from chemo-naive, initial diagnostic biopsies. Conversely, the NEUROD1-dominant SCLC-N subtype, which is frequently cited as the second most prevalent variant in transcriptomic literature, was restricted to 1% (*n* = 1) of the study cohort. This low prevalence may correlate with the histopathological composition of the study population, which consisted entirely of pure SCLC cases. NEUROD1 expression has been documented to be significantly higher in combined SCLC architectures than in pure small cell carcinomas, highlighting a potential pathological distinction [21].

Conversely, the POU2F3-dominant SCLC-P subtype—which correlates with chemosensory tuft cell signatures in the respiratory tract and typically exhibits non-neuroendocrine characteristics—was not documented in a mono-dominant form within this cohort (0%) [11]. However, 5% of patients exhibited a co-dominant SCLC-AP phenotype, marked by concurrent baseline immunoreactivity for both ASCL1 and POU2F3. Early transcriptomic and proteomic models suggested that POU2F3 expression was mutually exclusive with ASCL1 and NEUROD1 profiles [9,22]. Nonetheless, recent multi-center investigations demonstrate that these lineage markers can be co-expressed, challenging the strict boundaries of traditional mutual exclusivity models. For instance, POU2F3 positivity has been reported in 24.6% of ASCL1-dominant cases, indicating a partial overlap between these transcription factors [23]. Similarly, synchronous high-level expression of ASCL1 and POU2F3 within identical geographical tumor regions has been documented in metastatic lesions [17]. The documentation of a 5% co-dominant SCLC-AP phenotype at the protein level in the current evaluation confirms that POU2F3 activation is not strictly restricted to non-neuroendocrine phenotypes. It can be expressed concurrently with classical neuroendocrine drivers, reflecting asymmetric intratumoral subclonal heterogeneity across treatment-naive populations [24].

Similarly, the co-dominant SCLC-AN phenotype, observed in 9% of the study cohort, supports this transcriptional heterogeneity [1]. In our quantitative analyses, the statistically significant higher quantitative NEUROD1 expression levels documented in the SCLC-AN group compared with the mono-dominant SCLC-A subgroup (*p* < 0.001) suggest that these findings reflect a dynamic continuum between SCLC molecular variants rather than coincidental focal staining. Literature models demonstrate that SCLC cells can transition from an ASCL1-high state to a NEUROD1-high state [24]. Consequently, the SCLC-AN phenotype may represent intermediate subclones captured during this dynamic phenotypic transition phase [17].

YAP1, initially proposed as a fourth independent molecular lineage driver (SCLC-Y), is not currently validated as a tumor cell-intrinsic driver in published series [23]. The baseline finding that YAP1 expression, documented in 24% of our cohort, exhibited no statistically significant variation across stratified molecular variants (*p* = 0.695) supports the consensus that YAP1 does not operate as an independent lineage driver in SCLC [16]. Quantitative transcriptomic and single-cell evaluations have shown that YAP1 is primarily expressed within normal epithelial and stromal cell populations of the tumor microenvironment rather than malignant epithelial cells [9,13]. These dynamics indicate that YAP1 may serve as a surrogate marker of a neuroendocrine-low microenvironment or mesenchymal transformation rather than an autonomous oncogenic lineage driver [9]. Our Spearman correlation analysis revealed a weak positive correlation between POU2F3 and YAP1 expressions (ρ = 0.16) and a minor negative correlation trend between ASCL1 and YAP1 (ρ = −0.13) (Figure 5), aligning with current structural models in the literature.

The co-dominant distributions of 9% SCLC-AN and 5% SCLC-AP documented in this evaluation highlight the intratumoral heterogeneity characterizing SCLC [25]. Given that SCLC is primarily diagnosed utilizing small bronchoscopic or needle biopsies in clinical practice [3], this subclonal mosaicism becomes clinically paramount. A single small biopsy specimen may be subject to sampling limitations, potentially failing to comprehensively capture the dynamic proteomic landscape of the tumor or establish a definitive dominant lineage driver [16,26]. Published models emphasize that immunophenotypic discordance can manifest across disparate foci within resection specimens or between primary tumor sites and synchronous distant metastases, indicating that limited tissue sampling may underrepresent the plastic architecture of the disease [17]. Beyond this clinical independence, the subclonal intratumoral heterogeneity quantified via IHC highlights operational constraints when interpreting isolated biopsy fragments. SCLC is primarily diagnosed using small-needle or bronchoscopic biopsies in clinical practice; therefore, this asymmetric molecular mosaicism and the presence of co-dominant hybrid phenotypes, such as SCLC-AN and SCLC-AP, indicate that sampling limitations may fail to comprehensively capture the dynamic molecular landscape of the tumor [13]. The underlying biological plasticity of SCLC presents challenges for integrating a static classification framework based strictly on discrete proteomic thresholds into routine clinical algorithms. Consequently, the proposed IHC subtyping framework should be interpreted as a flexible tracking model rather than an absolute taxonomic entity.

Furthermore, the treatment-adjusted parsimonious multivariate Cox regression framework demonstrated that individual proteomic molecular subtype classifications and quantitative transcription factor continuous expression profiles did not exert an autonomous independent prognostic effect on overall survival clinical endpoints (Figure 9). These outcomes align with consensus data suggesting that individual SCLC transcriptional lineage variants do not operate as independent prognostic factors for overall survival prediction across unselected populations [26,27]. Conversely, within this adjusted architecture, traditional extensive-stage disease (HR = 3.801, 95% CI: 1.669–8.657, *p* = 0.001) and the clinical presence of synchronous bone metastases at initial presentation (HR = 1.968, 95% CI: 1.074–3.604, *p* = 0.028) were validated as the only robust independent predictors of poor overall survival endpoints across the cohort [27].

Our clinicopathological evaluations demonstrated no statistically signficant associations between molecular variants and baseline demographic characteristics (chronological age, sex, smoking history) or distinct metastasis patterns, including liver, bone, and adrenal involvement (Table 1) [9]. This uniform distribution may reflect the baseline biological aggressiveness characterizing SCLC, irrespective of individual transcriptional subtypes [28]. Although published data suggest that NEUROD1-dominant or mixed phenotypes may differ in their organotropism toward brain metastasis or central nervous system progression kinetics [17], our cohort demonstrated no evidence of topographic clustering. This finding may be related to the sample dynamics of our cohort, or it may suggest that universal *TP53* and *RB1* co-inactivation in the pathogenesis of SCLC constitutes a dominant aggressive background across all lineage variants [16]. Ultimately, these outcomes confirm that transcriptional markers quantified via IHC alone lack autonomous prognostic value for overall survival clinical endpoints. Advanced clinical stage and synchronous bone metastases remain the primary independent clinical predictors of survival across the study population.

Furthermore, data derived from the therapeutic exploratory subcohort (*n* = 19) receiving first-line platinum–etoposide concurrently with atezolizumab provide baseline observations regarding whether these IHC-based immunophenotypic profiles offer predictive translational insights [10]. Due to sample size constraints within individual subclonal lines, patients were dichotomized into two categories based on neuroendocrine marker baseline accumulation [7]; all patients within the low neuroendocrine level group (NE-low/Non-NE, *n* = 3) achieved an objective clinical response (Figure 10A). However, the limited sample size (*n* = 19) of this specific therapeutic subgroup reduces the statistical power of these survival assessments. Consequently, the 100% objective response rate documented within the NE-low phenotypic cohort represents a preliminary baseline observation that cannot be extrapolated to the broader unselected SCLC population, highlighting the need for prospective multi-center validation cohorts before general clinical implementation.

In the Kaplan–Meier analysis, the median overall survival was 49.4 months for the NE-low/Non-NE group, whereas a numerically shorter median overall survival of 21.4 months was documented for the NE-high group (*n* = 16). Although this difference was not statistically significant, a numerically longer median overall survival was observed in the NE-low/Non-NE group (log-rank *p* = 0.080) (Figure 10B). This finding may be related to the limited sample size of this retrospective analysis and potential fluctuations in median estimations driven by individual long-term survivors within this specific therapeutic subcohort. Consequently, these limited numerical metrics should be interpreted not as definitive clinical conclusions, but as preliminary observations warranting future longitudinal tracking of potential relationships between neuroendocrine driver distribution and immune checkpoint inhibitor response across larger, multi-center cohorts. Indeed, the therapeutic potential for neuroendocrine-low variants to derive survival benefits from novel chemoimmunotherapy regimens remains an active topic of investigation across the literature [12].

Despite the potential clinical and translational implications of these findings, several limitations stemming from the pilot sample size should be acknowledged. First, this investigation is characterized by a retrospective, single-center design. Second, the therapeutic subcohort receiving atezolizumab-based chemoimmunotherapy is relatively small (*n* = 19), with a highly restricted sample size within the NE-low phenotypic group (*n* = 3). Third, a primary limitation involves the unavailability of cytotoxic T-cell marker immunohistochemical evaluations, such as CD8 staining, which prevented the definitive verification of whether the identified quadruple-negative SCLC-QN variants correlate with the SCLC-inflamed (SCLC-I) immunophenotype reported in the literature [12]. Similarly, the inability to immunohistochemically quantify DLL3 expression, a key translational therapeutic target across classical SCLC subtypes, represents a further operational constraint [8]. Finally, while the quantitative IHC subtyping method utilized here offers a cost-effective and pragmatic alternative in routine laboratory workflows, the comprehensive gene-mapping capabilities of transcriptomic sequencing (RNA-seq) platforms covering thousands of targets remain distinct [9]. It should be noted that the survival timelines observed within this pilot subcohort are numerically longer than the historical overall survival data (12–13 months) reported across landmark phase 3 clinical trial datasets in the literature [29,30].

## 5. Conclusions

In conclusion, this pilot investigation characterizes the proteomic landscape of SCLC by evaluating a pragmatic and cost-effective four-marker IHC subtyping framework (ASCL1, NEUROD1, POU2F3, and YAP1) designed for routine pathology workflows. The identification of co-dominant hybrid phenotypes, such as SCLC-AN and SCLC-AP, supports the paradigm of underlying subclonal intratumoral heterogeneity and lineage plasticity in SCLC. Although these transcription factors did not exert an independent prognostic effect on overall survival clinical endpoints within the full study population, observations within the systemic therapeutic subcohort indicate that these proteomic profiles may provide predictive translational insights regarding chemoimmunotherapy outcomes. Ultimately, if validated by future large-scale, prospective, and multi-center clinical trials, this quantitative IHC-based subtyping strategy could facilitate the transition of SCLC management away from uniform staging protocols and toward biomarker-driven personalized therapeutic algorithms.

## Figures and Tables

**Table 1:** Baseline clinicopathological characteristics, distant metastatic patterns, and master transcription factor expression profiles across IHC-defined SCLC molecular subtypes (*n* = 100).

Variable	SCLC-A (*n* = 83)	SCLC-N (*n* = 1)	SCLC-AN (*n* = 9)	SCLC-QN (*n* = 2)	SCLC-AP (*n* = 5)	*p*-Value
**Demographic and clinical characteristics**						
Age at diagnosis (years), Mean ± SD	62.1 ± 8.6	60.0 ± NA	62.6 ± 8.4	58.5 ± 27.6	63.2 ± 2.9	0.974
Sex (Male), *n* (%)	63 (75.9)	1 (100)	6 (66.7)	2 (100)	5 (100)	0.560
Comorbidity (Present), *n* (%)	42 (50.6)	0 (0)	5 (55.6)	1 (50.0)	4 (80.0)	0.597
VALG Stage (Extensive), *n* (%)	58 (69.9)	1 (100)	6 (66.7)	1 (50.0)	3 (60.0)	0.905
Smoking history (Yes), *n* (%)	54 (65.9) *	0 (0)	8 (88.9)	2 (100)	4 (80.0)	0.247
**Categorical Lineage Marker Positivity** *						
ASCL1 (Positive), *n* (%)	80 (96.4)	0 (0)	6 (66.7)	0 (0)	5 (100)	**<0.001**
YAP1 (Positive), *n* (%)	19 (22.9)	0 (0)	3 (33.3)	0 (0)	2 (40.0)	0.711
NEUROD1 (Positive), *n* (%)	29 (34.9)	1 (100)	9 (100)	0 (0)	1 (20.0)	**0.001**
POU2F3 (Positive), *n* (%)	19 (22.9)	0 (0)	1 (11.1)	0 (0)	5 (100)	**0.002**
**Continuous Quantitative Biomarker Levels (%)**						
ASCL1 expression, Median [IQR]	95 [20.00]	0 [0.00]	70 [65.00]	0.00 [0.00]	100 [5.00]	**0.004** †
YAP1 expression, Median [IQR]	0 [0.00]	0 [0.00]	0 [1.00]	0.00 [0.00]	0 [3.00]	0.695 †
NEUROD1 expression, Median [IQR]	0 [10.00]	40 [0.00]	60 [70.00]	0.00 [0.00]	0 [0.00]	**<0.001** †
POU2F3 expression, Median [IQR]	0 [0.00]	0 [0.00]	0 [0.00]	0.00 [0.00]	80 [35.00]	**<0.001** †
**Synchronous Distant Metastatic Patterns** *						
Liver metastasis, *n* (%)	25 (30.1)	0 (0)	3 (33.3)	0 (0)	3 (60.0)	0.498
Brain metastasis (at presentation), *n* (%)	24 (28.9)	0 (0)	1 (11.1)	0 (0)	0 (0)	0.371
Bone metastasis, *n* (%)	38 (45.8)	0 (0)	4 (44.4)	0 (0)	1 (20.0)	0.461
Adrenal gland metastasis, *n* (%)	14 (16.9)	0 (0)	1 (11.1)	0 (0)	0 (0)	0.782
Brain metastasis during course, *n* (%)	27 (32.5)	0 (0)	1 (11.1)	1 (50.0)	0 (0)	0.311
**Longitudinal Limited-Stage Recurrence** ‡						
Recurrence event, *n* (%)	18 (72.0) **	0 (NA) §	1 (33.3) **	1 (100) **	2 (100) **	0.487

Data are presented as frequency (percentage), *n* (%), for categorical parameters, mean ± standard deviation (SD) for the normally distributed continuous age variable, and median [interquartile range, IQR] for non-normally distributed protein continuous percentages. Bolded *p*-values indicate statistical significance (*p* < 0.05). SCLC, small-cell lung cancer; VALG, Veterans Administration Lung Study Group; NA, not applicable. * Evaluated across groups using the Pearson chi-square (χ^2^) test, or Fisher’s exact test when expected cell frequencies were less than 5 to maintain statistical integrity within limited cohorts. ** explicitly clarify the adjusted baseline denominator, denoting that the categorical percentages for this longitudinal recurrence axis are calculated strictly within the active limited-stage patient subsets (*n* = 25) rather than the entire cohort. † Evaluated across groups using the nonparametric Kruskal–Wallis test for non-normally distributed continuous quantitative immunoreactivity percentages. ‡ Subgroup evaluation restricted strictly to patients presenting with limited-stage disease at baseline. This section sign (§) is identical in meaning to the double dagger (†) or single/triple indicators below, explicitly denoting that this specific sub-evaluation is restricted strictly to patients presenting with limited-stage disease at baseline. *Note on Lineage Marker Positivity:* Apparent discrepancies between categorical positivity and continuous median expression across individual columns (e.g., NEUROD1 positivity within the SCLC-A subset) are mathematically driven by the strict application of predefined asymmetric multi-marker clinical cut-offs (≥50% for ASCL1, ≥5% for NEUROD1, ≥1% for POU2F3, and >0% for YAP1) rather than a linear continuous correlation across the cohort.

**Table 2:** Univariate Cox proportional hazards regression analysis of lineage transcription factor expression profiles for overall survival across the small-cell lung cancer cohort (*n* = 100).

Variable	Hazard Ratio (HR)	95% Confidence Interval (CI)	*p*-Value
**ASCL1 expression (%)**	1.37	0.59–3.16	0.459
**NEUROD1 expression (%)**	1.02	0.64–1.62	0.946
**POU2F3 expression (%)**	0.99	0.59–1.64	0.956
**YAP1 expression (%)**	0.94	0.56–1.59	0.823

*Footnote:* OS, overall survival; HR, hazard ratio; CI, confidence interval. Continuous transcription factor percentages were evaluated utilizing univariate Cox proportional hazards regression models to assess baseline risk dimensions. Statistical significance was evaluated at the conventional alpha level (*p* < 0.05).

## Data Availability

The data presented in this study are available on request from the corresponding author.

## Supplementary Materials

The following supporting information can be downloaded at: https://www.mdpi.com/article/10.3390/jcm15166415/s1. Supplementary Table S1. Baseline clinical, demographic, and procedural characteristics of the study cohort (*n* = 100). Supplementary Table S2. Secondary immunohistochemical marker profiles, longitudinal recurrence rates, and clinical statistical parameters stratified across small cell lung cancer molecular subtypes (*n* = 100).
