# Supplementary material for: Quantitative Immunohistochemical Landscapes and Chemoimmunotherapy Outcomes of ASCL1, NEUROD1, POU2F3, and YAP1 in Pure Small-Cell Lung Cancer: A Pilot Study
Source: J Clin Med. 2026 Aug 19;15(16):6415. doi: 10.3390/jcm15166415 (PMC13513695; doi:10.3390/jcm15166415)
Supplement: Supplementary file 1 [file jcm-15-06415-s001.zip › jcm-4477550-supplementary.pdf]

Supplementary Table S1. Baseline clinical, demographic, and procedural characteristics of the study cohort (n = 100).

**Characteristic**	**Category**	**Value (n = 100)**
**Age at Diagnosis (years)**	Mean ± SD (Range)	62.1 ± 8.7 (33–78)
**Sex**	Male, n (%)	77 (77.0)
	Female, n (%)	23 (23.0)
**Smoking Status**	Yes, n (%)	68 (68.0)
	No, n (%)	32 (32.0)
**Comorbidities**	Present, *n* (%)	52 (52.0%)
	Absent, *n* (%)	48 (48.0%)
VALG Stage	Limited, n(%)	31 (31.0%)
	Extensive, n(%)	69 (69.0%)
Distant Metastasis Sites*	Bones, *n* (%)	43 (43.0%)
	Liver, *n* (%)	31 (31.0%)
	Brain, *n* (%)	29 (29.0%)
	Adrenal Glands, *n* (%)	15 (15.0%)
**Primary Biopsy Focus**	Lung (Bronchoscopic), *n* (%)	71 (71.0%)

Characteristic	Category	Value (n = 100)
	Mediastinal Lymph Nodes, *n* (%)	11 (11.0%)
	Liver, *n* (%)	8 (8.0%)
	Pleura, *n* (%)	3 (3.0%)
	Bone, *n* (%)	2 (2.0%)
	Other Rare Metastatic Foci, *n* (%)	5 (5.0%)

Footnotes: SCLC, small cell lung cancer; VALG, Veterans Administration Lung Study Group; SD, standard deviation.
* Percentages for anatomical distant metastasis patterns do not sum to 100% due to patients presenting with multiple synchronous metastatic sites.

Supplementary Results: Statistical Evaluations of Marker Profiles

Categorical and Quantitative Analyses of Lineage Transcription Factors

ASCL1 categorical expression: ASCL1 positivity rates demonstrated statistically significant variations across SCLC molecular subtypes ($\chi^2 = 45.1$, df = 4, $p < 0.001$). Post hoc standardized residual analysis indicated that the ASCL1 positivity rate within the mono-dominant SCLC-A group was higher than expected (standardized residual = 4.50). In contrast, ASCL1 negativity rates were significantly higher than expected within the SCLC-N (standardized residual = −3.00), SCLC-AN (standardized residual = −2.10), and quadruple-negative SCLC-QN (standardized residual = −4.50) cohorts. The co-dominant SCLC-P and SCLC-AP groups did not deviate significantly from expected values (all standardized residuals $|\text{residual}| < 2.0$, $p > 0.05$).

NEUROD1 categorical expression: The NEUROD1 positivity rate differed significantly across the stratified molecular subsets ($\chi^2 = 18.1$, df = 4, $p = 0.001$). Post hoc analysis demonstrated that the NEUROD1 positivity rate was significantly higher than expected within the co-dominant SCLC-AN subgroup (standardized residual = 3.85), whereas it was significantly lower than expected within the mono-dominant SCLC-A subgroup (standardized residual = −2.28).

POU2F3 categorical expression: POU2F3 positivity rates demonstrated significant differences across subgroups ($\chi^2 = 17.1$, df = 4, p = 0.002). Post hoc residual monitoring showed that the POU2F3 positivity rate was significantly higher than expected exclusively within the co-dominant SCLC-AP cohort (standardized residual = 3.97). In all other molecular subgroups, POU2F3 positivity rates did not deviate significantly from expected distributions (all standardized residuals |residual| < 2.0, p > 0.05).

YAP1 categorical expression: No statistically significant differences were documented among the molecular variants regarding categorical YAP1 positivity rates (p = 0.711).

Quantitative continuous biomarker comparisons (Kruskal–Wallis and Post Hoc Evaluations):

- **Continuous ASCL1 percentage:** Quantitative baseline ASCL1 expression percentages differed significantly across the molecular subtypes ($\chi^2 = 15.64$, df = 4, p = 0.004). However, post hoc pairwise comparisons utilizing the Dwass–Steel–Critchlow–Fligner (DSCF) method demonstrated no statistically significant autonomous differences between individual subgroup pairs (all p > 0.05).
- **Continuous NEUROD1 percentage:** Quantitative NEUROD1 expression levels differed significantly across the subsets ($\chi^2 = 23.08$, df = 4, p < 0.001). Post hoc pairwise DSCF analysis verified that the mono-dominant SCLC-A cohort exhibited significantly lower continuous NEUROD1 levels compared with the co-dominant SCLC-AN subgroup (W = 6.210, p < 0.001). No significant differences were observed between other subtype pairs (all p > 0.05).
- **Continuous POU2F3 percentage:** Quantitative POU2F3 expression percentages demonstrated significant variations across the cohorts ($\chi^2 = 25.33$, df = 4, p < 0.001). Post hoc pairwise evaluations via the DSCF method unmasked the following significant links: the SCLC-A cohort exhibited significantly lower continuous POU2F3 expression levels than the co-dominant SCLC-AP group (W = 6.684, p < 0.001), and the SCLC-AN subgroup demonstrated significantly lower continuous POU2F3 levels compared with the co-dominant SCLC-AP cohort (W = 4.698, p = 0.008).
- **Continuous YAP1 percentage:** Continuous quantitative YAP1 expression percentages showed a uniform distribution and did not differ significantly across the molecular subsets ($\chi^2 = 2.22$, df = 4, p = 0.695).

Evaluations of Conventional Thoracic Pathological and Neuroendocrine Markers Quantitative Thyroid Transcription Factor-1 (TTF-1) expression demonstrated a borderline association with the molecular subtype distribution (p = 0.054), marking a numerical decline within non-classical variants. Conversely, no significant differences were observed across subgroups regarding categorical chromogranin A expression or synaptophysin positivity (p =

0.536). The total cumulative neuroendocrine marker score was statistically indistinguishable and uniform across all phenotypic cohorts (p = 0.962). Although categorical CD56 positivity demonstrated a statistical difference across the subsets (p = 0.002), this sub-evaluation was characterized by a high proportion of missing archival data (92% missing data across the cohort); therefore, this baseline finding should be interpreted with caution.

Supplementary Table S2. Secondary immunohistochemical marker profiles, longitudinal recurrence rates, and clinical statistical parameters stratified across small cell lung cancer molecular subtypes (n = 100).

**Variable**	**SCLC-A (n = 83)**	**SCLC-N (n = 1)**	**SCLC-AN (n = 9)**	**SCLC-QN (n = 2)**	**SCLC-AP (n = 5)**	**Test Metric / Post hoc Evaluation**	**p-value**
**Pathological Markers & Recurrence**							
**TTF-1** Positive, n (%)	78 (94.0)	1 (100.0)	8 (88.9)	2 (100.0)	3 (60.0)	Chi-square analysis	0.054
**Synaptophysin** Positive, n (%)	30 (36.1)	1 (100.0)	9 (100.0)	2 (100.0)	1 (20.0)	Chi-square analysis	0.536
**CD56** Positive, n (%)	6 (100.0)	0 (0.0)	0 (0.0)	0 (0.0)	2 (100.0)	Chi-square analysis *(92% missing data across cohort)*	**0.002**
**Total NE Marker Score,** n (%)	78 (96.3)	1 (100)	9 (100)	2 (100)	5 (100)	Integrated NE index	0.962
**Recurrence** (Limited Stage), n (%)	18 (72.0)	0 (0.0)	1 (33.3)	1 (100)	2 (100)	Restricted subset analysis	NA

**Metastasis during course**, n (%)	27 (32.5)	0 (0.0)	1 (11.1)	1 (50.0)	0 (0.0)	Longitudinal brain metastasis rates	0.311
**Post-hoc Residuals & Pairwise Tests**							
ASCL1 (Categorical)	—	—	—	—	—	SCLC-A: Standardized residual = 4.50 *(higher than expected)*<br>SCLC-N, AN, QN: Standardized residual < −2.00 *(lower than expected)*	**<0.001**
ASCL1 (Continuous %)	—	—	—	—	—	DSCF pairwise comparisons (All pairs $p > 0.05$)	**0.004**
NEUROD1 (Categorical)	—	—	—	—	—	SCLC-AN: Standardized residual = 3.85 *(higher than expected)*<br>SCLC-A: Standardized residual = −2.28 *(lower than expected)*	**0.001**
NEUROD1 (Continuous %)	—	—	—	—	—	SCLC-A vs. SCLC-AN *($W = 6.210$, $p < 0.001$)*	**<0.001**
POU2F3 (Categorical)	—	—	—	—	—	SCLC-AP: Standardized residual = 3.97 *(higher than expected)*	**0.002**
POU2F3 (Continuous %)	—	—	—	—	—	SCLC-A vs. SCLC-AP *($W = 6.684$, $p < 0.001$)*	**<0.001**

	SCLC-AN vs. SCLC-AP *(W = 4.698, p = 0.008)*	
**YAP1** (Categorical)	Subgroup comparison	**0.711**
**YAP1** (Continuous %)	Subgroup comparison	**0.695**

Footnote: SCLC, small cell lung cancer; NE, neuroendocrine; DSCF, Dwass–Steel–Critchlow–Fligner post hoc test; *W*, DSCF test statistic; NA, not applicable. Bolded p-values denote statistical significance ($p < 0.05$).
*Note: Chromogranin A metrics and CD56 pairwise comparisons were omitted from formal statistical hazard modeling due to a high proportion of missing retrospective clinical data across the cohort.
